# Influence of Malnutrition on Adverse Outcome in Children with Confirmed or Probable Viral Encephalitis: A Prospective Observational Study

**DOI:** 10.1155/2015/407473

**Published:** 2015-01-28

**Authors:** Priyanka Singh, Girish C. Bhatt, Vijay Singh, K. P. Kushwaha, Mahima Mittal, Anita Mehta, Bhoopendra Sharma, Abhijit P. Pakhare, Abhishek Kumar

**Affiliations:** ^1^Department of Pediatrics, BRD Medical College, Gorakhpur, India; ^2^Department of Pediatrics, All India Institute of Medical Sciences (AIIMS), Room No. 10, OPD Block, Saket Nagar, Bhopal, Madhya Pradesh 462024, India; ^3^Department of Community and Family Medicine, AIIMS, Bhopal, India

## Abstract

A prospective observational study was conducted in a tertiary care teaching hospital from August 2008 to August 2009 to explore the independent predictors of adverse outcome in the patients with confirmed/probable viral encephalitis. The primary outcome variable was the incidence of *adverse outcomes* defined as death or severe neurological deficit such as loss of speech, motor deficits, behavioural problems, blindness, and cognitive impairment. Patients with confirmed or probable viral encephalitis were classified into two groups based on their *Z*-score of weight-for-age as per WHO growth charts. *Group I.* Patients with confirmed or probable viral encephalitis with weight-for-age (W/A) *Z*-scores below −2SD were classified as undernourished. *Group II.* Patients with confirmed or probable viral encephalitis were classified as having normal nutritional status (weight-for-age *Z*-score >−2SD). A total of 114 patients were classified as confirmed or probable viral encephalitis based on detailed investigations. On multivariate logistic regression, undernutrition (adjusted OR: 5.05; 95% CI: 1.92 to 13.44) and requirement of ventilation (adjusted OR: 6.75; 95% CI: 3.63 to 77.34) were independent predictors of adverse outcomes in these patients. Thus, the results from our study highlight that the association between undernutrition and adverse outcome could be extended to the patients with confirmed/probable viral encephalitis.

## 1. Introduction

A wide range of viruses are implicated as a cause of acute viral encephalitis either in sporadic or in outbreak forms. Enteroviruses, flaviviruses, and herpes simplex, Chandipura, and West Nile viruses are some of the common causes of viral encephalitis [1–3]. Eastern Uttar Pradesh region of India is facing regular outbreaks of the encephalitis since 1978. Children below the age of 14 years were the most vulnerable population and despite mass JE vaccination following this epidemic, the death toll continued to rise. A change in the clinical pattern of encephalitis was observed in this region with JE positivity of about 5–17% [4]. Isolates from 306 patients with acute encephalitis during an outbreak of viral encephalitis in northern India identified enterovirus in about 21.6 percent of cases. Further sequencing and phylogenetic analyses of PCR products from 89.3% specimens showed similarity with EV-89 and EV-76 sequences [5].

A potential biochemical mechanism that could adversely affect seizure threshold (in patients with epilepsy), particularly the effect of malnutrition on inhibitory neurotransmitter and electrolyte, has been worked out where supportive evidence from animal research and epidemiological findings in children has been discussed [6]. A lot of research has been done in the past showing the interrelationship between poor nutritional status, infection, and immunity but no such work has been published regarding viral encephalitis or other infectious diseases. A recent study from India by Bhargava et al. [7] has shown a high prevalence (more than 85%) of undernutrition in patients with pulmonary tuberculosis at diagnosis and an increased risk of death among patients with undernutrition. However, there are no global data regarding the effect of undernutrition on the outcome of patients with viral encephalitis. Therefore, we conducted this study to find out an association between the status of nutrition of encephalitis patients and the clinical outcomes of the disease.

## 2. Methods

### 2.1. Design and Study Population

This prospective observational study was performed in a tertiary care centre of north India from August 2008 to August 2009. The study protocol was approved by institutional ethics committee. After obtaining written consent from parents, all consecutive patients, aged 2 months to 10 years, admitted to a tertiary care teaching hospital* with* AES [8] characterized by pleocytosis, an absence of bacteria upon culture of CSF, and some or all of the following features, fever, sensorium changes (such as confusion, disorientation, drowsiness, stupor, coma, convulsions, and abnormal behavior), ataxia, limb paralysis, hemiplegia, and specific cranial nerve dysfunction* plus* confirmed or probable viral etiology, were enrolled for the study. A detailed examination was done in a precoded performa and various samples were sent for microbiological investigations. For confirmation of viral etiological CSF, serum, throat swab, and stool samples were tested. CSF was also subjected to nested RTPCR or real-time PCR for detecting presence of enterovirus or JEV genome. All the samples were stored at −20°C before sending them to the National Institute of Virology (NIV). Based on these investigations patients were divided into the following.

*Patients with Confirmed Viral Etiological Agents.* Those patients with positive CSF PCR for JEV or enteroviruses and/or IgM antibodies against JEV in CSF were grouped as confirmed viral encephalitis.

*Probable Viral Etiological Agents.* Those patients with presence of enterovirus in stool or throat samples and absence of other viruses in CSF were classified as probable viral etiological agents.

Based on CSF examination, bacterial culture, and other examinations, patients with bacterial meningitis, tuberculous meningitis, cerebral malaria, and typhoid fever were excluded. Causes of the consciousness level caused by factors other than infections (such as hypoglycaemia, haemorrhage, infarction, and other metabolic causes) were excluded from the study. Patients with suspected viral encephalitis in which no etiological agent could be identified were also excluded from the study.

Further, patients with confirmed or probable viral encephalitis were classified into two groups based on their *Z*-score of weight-for-age as per World Health Organization (WHO) charts [9].

*Group I.* Patients with confirmed or probable viral encephalitis with weight-for-age (W/A) *Z*-scores below −2SD were classified as ill-nourished [9].

*Group II.* Patients with confirmed or probable viral encephalitis with normal nutritional status (weight-for-age *Z*-score >−2SD) were classified as normally nourished.

### 2.2. Outcome Variables

The primary outcome variable was the incidence of adverse outcomes defined as death or severe neurological deficit such as loss of speech, motor deficits, behavioural problems, blindness, and cognitive impairment. We also evaluated other sociodemographic and clinical factors for association with adverse outcome

The factors which were evaluated in our study were prehospital factors such as rural or urban background, socioeconomic status, time interval between onset of symptoms and hospital arrival time, and total duration of hospital stay. Factors at admission which were evaluated include demographic characteristics of the population such as age, sex, gender, presence of undernutrition, clinical features, and investigations.

All the patients were followed up until death or discharge, whichever came first. All the baseline characteristics were collected at the time of admission. For determining risk factors we compared the predefined factors of the children who died or had severe neurological deficit during hospital stay.

Patients in both groups received treatment as per the standard protocol for encephalitis while patients in undernutrition group received recommended treatment for undernutrition [8].

### 2.3. Statistical Analysis

Statistical analysis was done by using SPSS-21 software. Sociodemographic and clinical characteristics were described using counts and proportions for categorical variables and mean and standard deviation for numerical variables. Chi-square test was used to test difference among malnourished and normal groups. A *P* value of less than 0.05 was considered statistically significant. Univariate logistic regression analysis was performed to test association of various factors with outcome, namely, favourable versus adverse. Then statistically significant factors (Table 1) and certain known predictors like pallor, place residence, and hospitalization interval were also included in multivariate logistic regression model [4]. Stepwise forward conditional model was used for identifying independent predictors. Hosmer-Lemeshow Goodness of Fit test was used for testing fit of model.

## 3. Results

A total of 1286 patients were screened during this period and 114 patients were classified as confirmed or probable viral encephalitis based on detailed investigations. Out of these 80 patients were grouped as undernutrition (group I) and 34 patients were classified as normally nourished (group II). The mean age of presentation was 5.26 ± 2.5 years (mean ± standard deviation) in group 1 and 5 ± 2 years in group II. At the time of admission the statistically significant difference between two groups was found for variables, namely, swelling all over the body, pallor, edema, signs of dehydration, tachycardia, and raised blood urea present in undernutrition group (Table 1). A total of 54 patients were classified as confirmed JE, 32 patients as confirmed enteroviruses, and 28 patients as probable enterovirus encephalitis.

### 3.1. Complications and Outcome

A total of 24 patients expired during hospital stay, 21 in group I and 3 in group II. The most common complications present in these patients were aspiration pneumonia (12), gastrointestinal bleeding (11), congestive heart failure (8), peripheral vascular failure (8), and cerebral herniation (6). A total of 37 patients required ventilation, the most common indications being respiratory failure followed by shock and low Glasgow coma scale. The mean duration of ventilation was 34 (29) hours in both groups. Severe neurological deficit was present in 49 patients (41 in group I and 08 in group II). Thus incidence of adverse outcome in undernourished group was 62/80 (77.5%; 95% CI: 67.2 to 87.8) which was higher than that in normally nourished group [11/34 (32.4%; 95% CI: 19.1 to 49.2)].

### 3.2. Risk Factor for Poor Outcome

On univariate analysis for association of various factors with outcome categories, presence of malnutrition, edema, signs of meningeal irritation, gastrointestinal bleed, respiratory distress, and need for ventilator support were statistically significantly associated with adverse outcome (Tables 2 and 3). On multivariate analysis by forward conditional method, only presence of undernutrition (adjusted OR: 5.05; 95% CI: 1.92 to 13.44) and requirement of ventilation within 48 hours (adjusted OR: 6.75; 95% CI: 3.63 to 77.34) were independently associated with adverse outcomes. Hosmer-Lemeshow Goodness of Fit test was nonsignificant (*P* = 0.956) indicating fit of model, Omnibus Test of Model Coefficients on step 2 was significant (*P* = 0.001), and Nagelkerke R Square was 0.408.

## 4. Discussion

Our study found that undernutrition and requirement of ventilator support within 48 hours of admission are independent predictors of adverse outcome. To the best of our knowledge, the association between undernutrition and poor outcomes in patients with viral encephalitis has not been previously reported. A study on long term outcomes of Japanese encephalitis (JE) virus in central Sarawak, Malaysia, has shown poor perfusion, Glasgow coma scale ≤8, and more than two witnessed seizures as predictors of poor outcome at hospital discharge [10]. Other studies have shown younger age, higher body temperature, high white cell count in cerebrospinal fluid, and deep coma at hospital admission, altered sensorium, and focal neurologic deficit as predictor of poor outcomes in patients with JE [11, 12].

Previous studies have shown age, lower Glasgow coma scale (GCS) at admission, pallor, peripheral vascular failure, cerebral edema, status epileptics, thrombocytopenia, and requirement of ventilator support as independent predictors of mortality in the patients with viral encephalitis [4, 13, 14]. In a recent retrospective review of the patients with central nervous system infections which included patients with tuberculous meningitis, viral encephalitis, pyogenic meningitis, and fungal meningitis admitted to neurological critical care unit of a tertiary care centre, duration of hospital stay and mechanical ventilation were found to be independent predictors of mortality [15].

About 70% of the patients in our study had undernutrition (weight-for-age *Z*-score <−2SD). The prevalence of malnutrition in India is 43% [16] and Uttar Pradesh region is one of the worst regions affected with about 40% of its children being underweight and 56.8% stunted [17].

Nutritional status can be improved in the community by a number of interventions such as initiation of early breastfeeding and complementary feeding through nutrition counseling with some additional measures necessary in food insecure settings [18, 19]. The excess morbidity and mortality associated with undernutrition owed to the impairment of host defense mechanisms, which predisposes them to infectious diseases [20]. Thus, our study suggests that this association between undernutrition and adverse outcomes extends to the patients with viral encephalitis (JE and enteroviruses).

Requirement of ventilation within 48 hours of admission was found to be strongly associated with adverse outcome in our study. These findings are consistent with previous reports of critically ill patients with all cause encephalitis [13]. This finding indicates that physicians must assess the need for mechanical ventilation early with ongoing evaluation of the need for respiratory support [15].

Our study has certain limitations. As our primary objective was to explore risk factors associated with poor outcomes, considering feasibility of data collection in limited time period, we have accrued consecutive samples and no formal sample size calculations were done. As Japanese encephalitis and enteroviruses are the two most common etiological agents responsible for major outbreak of viral encephalitis in this region, risk factors for poor outcomes of patients suffering from these two organisms were included. Moreover, the evaluation of patients for chronic malnutrition and micronutrient status could not be done.

The strengths of our study are as follows: being the first study to show the association of undernutrition and poor outcomes in the patients with viral encephalitis; sampling of all consecutive patients coming to our institute which is only tertiary centre catering these patients, thus making selection bias less likely; inclusion of confirmed or probable viral encephalitis patients making results more disease specific.

This study has some important implications: undernutrition was highly prevalent in the patients with viral encephalitis and was associated with poor outcomes in these patients. It is an established fact that the economic condition of the family has a huge impact on the nutritional status of the children [18]. Malnutrition commonly affects all groups in a community, but infants and young children are most vulnerable because of their high nutritional requirements for growth and development. A comprehensive programme approach to improving complementary feeding practices including timely introduction of age-appropriate and hygienically prepared complementary foods, counseling for caregivers on feeding and care practices, optimal use of locally available foods, improving access to quality foods for poor families through social protection schemes and safety nets, and the provision of fortified foods and micronutrient [18, 19] is the need of the hour. Proper supportive care is the mainstay of treatment in cases of enterovirus or Japanese encephalitis as no specific treatment is presently available [21–24]. Further studies are required on long term follow-up and effect of nutritional intervention on the outcomes of the patients with viral encephalitis.

## 5. Conclusion

Our study found that undernutrition and requirement of ventilator support within 48 hours of admission are independent predictors of adverse outcome. Thus, the results from our study highlight that the association between undernutrition and adverse outcome could be extended to the patients with confirmed/probable viral encephalitis. Moreover, the results from the present study will be helpful for future design of treatment and preventive strategies of the disease.

## Figures and Tables

**Table 1:** Clinical and laboratory parameters in group I and group II.

Variables	Group I (*n* = 80)	Group II (*n* = 34)	*P* value
Clinical features			
Rashes	20 (25)	06 (17.64)	0.392
Swelling	37 (46.25)	08 (23.52)	**0.023**
Headache	14 (17.50)	04 (11.76)	0.442
Unconsciousness	58 (72.5)	25 (73.5)	0.910
Altered behavior	22 (27.5)	09 (26.5)	0.910
GTCS	59 (73.75)	23 (67.6)	
Focal seizures	10 (12.5)	07 (20.6)	0.537
No seizure	11 (13.75)	04 (11.76)	
Difficulty in breathing	19 (23.75)	07 (20.6)	0.713
Pallor	22 (27.5)	03 (8.82)	**0.027**
Pedal oedema	37 (46.3)	08 (23.52)	**0.023**
Signs of dehydration	20 (25.0)	None	**0.0007**
GCS score			
3–6	44 (55.0)	15 (44.2)	
7–10	19 (23.7)	11 (32.4)	0.529
>10	17 (21.3)	08 (23.5)	
Hepatomegaly	36 (45.0)	18 (52.9)	0.437
Splenomegaly	33 (41.25)	32 (94.12)	<0.001
Tachycardia	48 (60.0)	13 (38.2)	**0.033**
Laboratory investigations			
CSF cell count			0.074
6–100	68 (85)	24 (70.5)	
>100	12 (15)	10 (29.5)	
SGPT raised (15–40 U/L)	50 (62.5)	25 (73.3)	0.256
Blood urea raised (20–40 mg per 100 mL)	55 (68.7)	04 (11.76)	**0.0001**
ECG changes	20 (25)	09 (26.4)	0.868
Abnormal CT scan findings	6/33	03/12	0.613

**Table 2:** Univariate and multivariate logistic regression test to identify independent predictors of adverse outcome in patients of viral encephalitis.

^*^Univariate analysis			
Variable	Number (*n*)	Odds ratio (95% CI)	*P* value
Undernutrition	Yes 80 No 34	6.27 (2.6–15.10)	<0.001
Requirement of ventilation	Yes 37 No 77	19.93 (4.47–88.75)	<0.001
Rural Urban	94 20	1.8 (0.70–4.89)	0.216
Abdominal pain	Yes 19 No 95	3.88 (1.059–14.21)	0.0041
Splenomegaly	Yes 65 No 49	0.497 (0.225–1.094)	0.082
Congestive heart failure	Yes 21 No 93	2.21 (0.075–6.55)	0.152
Peripheral vascular failure	Yes 11 No 103	6.88 (0.85–55.83)	0.071
Requirement of ICU care	Yes 40 No 74	5.67 (2.13–15.10)	0.001
Edema	Yes 45 No 69	3.21 (1.38–7.48)	0.007
Signs of meningeal irritation	Yes 10 No 104	6.09 (0.74–49.92)	0.092
Aspiration pneumonia	Yes 15 No 99	4.60 (0.98–21.46)	0.053
Acute renal failure	Yes 25 No 89	1.74 (0.66–4.60)	0.260
Gastrointestinal bleeding	Yes 16 No 98	2.99 (0.80–11.17)	0.104
Prehospitalization interval	≤3 days 33 >3 days 81	0.63 (0.27–1.44)	0.278
Undernutrition by requirement of ventilation (interaction)		32.55 (4.24–249.8)	0.001

^#^Multivariate analysis			
Variable	Variable	Variable	Variable

Undernutrition	Yes 80 No 34	5.05 (1.9–13.44)	0.001
Requirement of ventilation	Yes 37 No 77	16.75 (3.63–77.34)	<0.001

CI: confidence interval; OR: odds ratio.

^*^Based on logistic regression test.

^#^Variables excluded after forward variable selection.

**Table 3:** Neurological deficit present in two groups at the time of discharge.

Residual neurological deficits	Group I		Group II		*P* value
	Number	%	Number	%	
Behavioral problem	41	51.25	06	17.6	0.0045
Involuntary movements	13	16.25	00	00	0.009
Hypertonia	11	13.75	00	00	0.03
Speech deficit	16	20.00	01	02.9	0.02
Abnormal gait	18	22.50	02	05.8	0.03
Hemiparesis	03	3.7	01	2.9	1.0

## References

[1] Glaser C. A., Gilliam S., Schnurr D. (2003). In search of encephalitis etiologies: diagnostic challenges in the California Encephalitis Project; 1998–2000. *Clinical Infectious Diseases*.

[2] Kennedy P. G. E. (2005). Viral encephalitis. *Journal of Neurology*.

[3] Kennedy P. G. E. (2004). Viral encephalitis: causes, differential diagnosis, and management. *Journal of Neurology, Neurosurgery & Psychiatry*.

[4] Bhatt G. C., Bondre V. P., Sapkal G. N. (2012). Changing clinico-laboratory profile of encephalitis patients in the eastern Uttar Pradesh region of India. *Tropical Doctor*.

[5] Sapkal G. N., Bondre V. P., Fulmali P. V. (2009). Enteroviruses in patients with acute encephalitis, Uttar Pradesh, India. *Emerging Infectious Diseases*.

[6] Hackett R., Iype T. (2001). Malnutrition and childhood epilepsy in developing countries. *Seizure*.

[7] Bhargava A., Chatterjee M., Jain Y. (2013). Nutritional status of adult patients with pulmonary tuberculosis in rural central India and its association with Mortality. *PLoS ONE*.

[8] WHO (2006). Recommended standards for surveillance of selected vaccine-preventable disease. http://www.who.int/vaccines-documents/DocsPDF06/843.pdf.

[9] The WHO growth chart-World Health Organization. http://www.who.int/childgrowth/publications/WHO_growth_charts.pdf.

[10] Mong H. O., Lewthwaite P., Boon F. L. (2008). The epidemiology, clinical features, and long-term prognosis of Japanese encephalitis in central Sarawak, Malaysia, 1997–2005. *Clinical Infectious Diseases*.

[11] Rayamajhi A., Singh R., Prasad R., Khanal B., Singhi S. (2006). Clinico-laboratory profile and outcome of Japanese encephalitis in Nepali children. *Annals of Tropical Paediatrics*.

[12] Luo D., Song J., Ying H., Yao R., Wang Z. (1995). Prognostic factors of early sequelae and fatal outcome of Japanese encephalitis. *The Southeast Asian Journal of Tropical Medicine and Public Health*.

[13] Thakur K. T., Motta M., Asemota A. O. (2013). Predictors of outcome in acute encephalitis. *Neurology*.

[14] Le V. T., Phan T. Q., Do Q. H. (2010). Viral etiology of encephalitis in children in southern Vietnam: results of a one-year prospective descriptive study. *PLoS Neglected Tropical Diseases*.

[15] Misra U. K., Kalita J., Bhoi S. K. (2014). Spectrum and outcome predictors of central nervous system infections in a neurological critical care unit in India: a retrospective review. *Transactions of the Royal Society of Tropical Medicine and Hygiene*.

[16] Progress for children report—a statistical review. http://www.unicef.org/india/media_3766.htm.

[17] UNICEF http://www.unicef.org/infobycountry/india.

[18] Children in India 2012—A statistical appraisal. http://mospi.nic.in/mospi_new/upload/Children_in_India_2012.pdf.

[19] UNICEF India-Nutrition http://www.unicef.org/india/nutrition.html.

[20] Melby P. C., Chandrasekar B., Zhao W., Coe J. E. (2001). The hamster as a model of human visceral leishmaniasis: progressive disease and impaired generation of nitric oxide in the face of a prominent Th1-like cytokine response. *The Journal of Immunology*.

[21] Kumar R., Tripathi P., Rizvi A. (2009). Effectiveness of one dose of SA 14-14-2 vaccine against Japanese encephalitis. *The New England Journal of Medicine*.

[22] Bhatt G. C., Sankar J., Kushwaha K. P. (2012). Use of intravenous immunoglobulin compared with standard therapy is associated with improved clinical outcomes in children with acute encephalitis syndrome complicated by myocarditis. *Pediatric Cardiology*.

[23] Kakkar M., Rogawski E. T., Abbas S. S. (2013). Acute encephalitis syndrome surveillance, Kushinagar District, Uttar Pradesh, India, 2011–2012. *Emerging Infectious Diseases*.

[24] Jain S., Patel B., Bhatt G. C. (2014). Enteroviral encephalitis in children: clinical features, pathophysiology, and treatment advances. *Pathogens and Global Health*.

